# Assessment of serum electrolytes and kidney function test for screening of chronic kidney disease among Ethiopian Public Health Institute staff members, Addis Ababa, Ethiopia

**DOI:** 10.1186/s12882-020-02166-0

**Published:** 2020-11-18

**Authors:** Meseret Derbew Molla, Maria Degef, Abebe Bekele, Zeleke Geto, Feyissa Challa, Tadesse Lejisa, Tigist Getahun, Meron Sileshi, Yosef Tolcha, Genet Ashebir, Daniel Seifu

**Affiliations:** 1 Department of Biochemistry, School of Medicine, College of Medicine and Health Sciences, University of Gondar, Gondar, Ethiopia; 2 Department of Medical Biochemistry, College of Health Sciences, Addis Ababa University, Addis Ababa, Ethiopia; 3 Ethiopian Public Health Institute, Addis Ababa, Ethiopia; 4 Department of Biochemistry, Division of Biomedical Sciences, University of Global Health Equity, Kigali, Rwanda

**Keywords:** Screening, GFR, CKD, And risk factors

## Abstract

**Background:**

Chronic kidney disease (CKD), which is characterized by its asymptomatic nature until an end stage, is one of the most common public health problems in the world. Thus, a regular checkup, especially for those individuals with high risk groups is inevitably important, and the screening has been done with laboratory findings. However, in developing countries, including Ethiopia screening for CKD are rarely done, and it is becoming common to hear sudden death from the kidney failure. Therefore, we aimed to screen serum electrolyte levels and estimated glomerular filtration rate (eGFR) among Ethiopian Public Health Institute (EPHI) staff members for an early detection of CKD and to identify the factors associated with it.

**Methods:**

A cross-sectional study was conducted from July 1 to October 28, 2018 among EPHI staff members. The level of serum creatinine and electrolytes were measured using COBAS 6000 analyzer. Then, eGFR was calculated using MDRD and CKD-EPI equations. Data analysis were done using SPSS version 20, and the factors associated with the outcome variable were assessed using logistic regression. *P* values < 0.05 were considered as statistically significant.

**Results:**

This study found that 3.6 and 1.9% of the study participants were at CKD stage II by MDRD and CKD-EPI equations, respectively. Out of the total study participants, 9.5% had hyperkalemia (serum potassium level > 5.0 mmol/L) and 8.5% had hypocalcemia (serum calcium level < 2.15 mmol/L). An older age (*P* = 0.006), high BMI (*P* = 0.045) and previous history of CVDs (*P* = 0.033) were found to be significantly associated factors with reduced glomerular filtration rate. Nine percent of the study participants were obese, 6.1% had family history of kidney failure, 18% self-reported history of hypertension, 3.4% diabetic and 5.3% had CVDs. About 51.2% of the study participants were males, and the majorities, (66%) of the study participants were found to be alcohol consumers.

**Conclusions:**

The prevalence of a stage II kidney disease was relatively low and none of the participants was under serious kidney disease (GFR < 60 mmol/min/1.73m^2^). An older age, high BMI and previous history of CVDs were significantly associated with reduced GFR. Hyperkalemia and hypokalemia were the major electrolyte disorders in the study participants.

## Background

Non-communicable diseases (NCDs) get global attention because of their rapid increase in the morbidity and mortality rates [1]. Of the total death from 2006 to 2016 in the globe, the majority (72.3%) was caused by NCDs with an increasing trend each year [2]. Even though cardiovascular diseases (CVDs) are the major causes of death among NCDs, CKD is also becoming one of the public health problems, especially in developing countries [3, 4]. Globally, the prevalence of CKD is estimated between 11 to 13% by which a stage III is the most common [5]. Several studies have reported that around 80% of CKD patients are found in developing countries with estimated prevalence of 14.3–36.1% with an annual end stage renal disease (ESRD) incidence rate of more than 500,000 patients [5, 6]. The average prevalence of CKD in Africa is 10.1% and the highest proportion (16.5%) is found to be in West/Central West Africa [4]. The prevalence of CKD in Sub-Saharan Africa countries is estimated to be 13.9% [6]. In this region, CKD is more prevalent in young productive age groups and the mortality rate is very high due to lack of an early screening and treatment facilities [6, 7].

The major obstacle to manage CKD is, its asymptomatic nature, the disease may not be recognized until end stage, and commonly called a silent killer [8]. Chronic kidney disease is a syndrome raised from different heterogeneous diseases is characterized by the persistent functional and/or structural abnormality of the kidney for more than 3 months [9, 10]. According to 2013 international guideline of CKD, the reduction of kidney functions which is detected by GFR < 60 ml/min per 1.73m^2^ with or without kidney damage that persist for at least 3 months regardless of the clinical diagnosis, and the underlining causes is defined as CKD [9]. The level of GFR can be estimated using several formulas; thus, the most studied and widely used formulas for adults are Cock Croft –Gault (C-G), Modification of Diet in Renal Disease (MDRD) and Chronic Kidney Disease Epidemiology Collaboration (CKD-EPI) equations [8]. These equations are calculated the level of eGFR using serum creatinine and other factors that affect the level of GFR such as age, sex and race [8, 11, 12]. For instance, In MDRD equation, GFR is estimated as: GFR (ml per minute per 1.73 m^2^) = 186 x (S_cr_ in mg/dL)^-1.154^ x (age in year)^-0.203^ x (0.742, if female) x (1.210, if black). Where, S_cr_ is serum creatinine [11]. In CKD-EPI equation, GFR is estimated as: GFR = 141 x min (Scr/k, 1) ^α^ x max (Scr/k, 1)^-1.209^ × 0.993^Age^× 1.018 (if female) × 1.159 (if black), where Scr is serum creatinine, k is 0.7 for females and 0.9 for males, α is − 0.329 for females and − 0.411 for males, min indicates the minimum of Scr/k or1, and max indicates the maximum of Scr/k or 1 [9]. Based on eGFR, CKD can be classified as; stage I (normal or high GFR > 90 ml/min per 1.73m^2^ with kidney damage indication), stage II (GFR between 60 and 90 ml/min per 1.73m^2^ with or without kidney damage indication), stage III (GFR between 30 and 59 ml/min per 1.73m^2^ with or without kidney damage indication), stage IV (GFR between 15 and 29 ml/min per 1.73m^2^ with or without kidney damage indication) and stage V or end stage renal failure (GFR < 15 ml/min per 1.73m^2^ with or without kidney damage indication) [9].

Globally, the stage III CKD is more prevalent than end-stage renal disease (ESRD) with an increasing rate over a time [4, 5]. Advanced CKD without the symptoms can lead to electrolyte disturbances since electrolytes homeostasis are maintained by the kidneys [13, 14]. Researchers have also found that the progression of CKD is one of the most common causes of the electrolyte disorders which increases the mortality rate, especially for those who are near to ESRD [13–16]. The most common electrolyte disorders associated with the renal failure are potassium, sodium, magnesium, phosphorus and calcium that can further lead to serious complications like a bone demineralization, muscle wasting, vascular calcification and even can result a death [13, 17]. Therefore, an early detection and treatment of CKD are very important to delay the development of ESRD and its complications [4]. But in developing countries, including Ethiopia screening for an early detection or identification of kidney diseases is rarely practiced. Thus, in Ethiopia, a hearing of kidney failure and/or death due to its complications becomes daily news. Furthermore, the management of ESRD and its complications is very difficult in developing countries due to several reasons such as the shortage of facilities and physicians, and unaffordability of costs to get the treatment abroad [4, 6]. Therefore, screening for an early detection of CKD patients is very important to minimize the economic burden of the population as well as to bring the quality care of the population. Thus far, there are limited numbers of studies conducted in Ethiopia, and the exact prevalence and incidence rate of CKD is not known. Therefore, in this study, we aimed to screen eGFR and serum electrolyte levels for an early detection of CKD, and to detect the associated factors of CKD among EPHI staff members.

## Methods

### Study design and setting

An institutional based cross-sectional study was conducted from July 1 to October 28, 2018 at EPHI located in Addis Ababa, capital city of Ethiopia. Ethiopian Public Health Institute is a well-known research institute in the country since 1995.

### Study participants and size

All volunteer staff members of the EPHI aged between 18 and 69 years who were working in the institute during the study period was participated in this study. The study participants were signed written informed consent before the start of data collection. The study participants were selected based on a convenient sampling technique and total of 412 study participants were included. The staff members of pregnant women, suffered from an acute infection and critically sick individuals were excluded from the study.

### Data collection and variables

Written informed consent was obtained from each study participants before running data collection. Data were collected using interviewer administered questionnaires. The questionnaires were prepared based on the previous related literatures and Kidney Disease Improving Global Outcomes (KDIGO) guidelines. It was developed by an English language since the source populations of the study were fully communicated with English. The questioner includes socio-demographic variables (sex, age, educational background and income status), behavioral variables (alcohol drinking status, cigarette smoking status, chat chewing status and traditional healer utilization practice), anthropometric variables (weight and height to calculate body mass index (BMI) and blood pressure) and clinically confirmed history of comorbid such as diabetes, hypertension, CVDs and kidney stone. Besides, the dependent outcomes (biochemical variables) were analyzed through the principle of standard measurement protocols. Data were collected by trained health professionals (Nurses, Public Health Officers and Physicians).

### Anthropometric measurements

The weight of the participants was measured in kilogram (kg) with light cloths and without wearing shoes, the height was measured in centimeter using height board without shoes in an upright position. Then, BMI was calculated by dividing weight in kg with height square; BMI = weight (kg)/height(m^2^) [18]. The blood pressure was measured according to WHO guideline using automated sphygmomanometer at the midpoint of the left arm after participants were rested for at least 5 min or 30 min for those who were taken hot drinks like coffee. Three blood pressure readings were taken for all participants, then the mean blood pressure was taken as true value [19]. The mean blood pressure ≤ 89/59 mmHg was defined as low/hypotension, between 90/60–139/89 mmHg was defined as normal and ≥ 140/90 mmHg was defined as high or hypertension by systolic/diastolic, respectively.

### Blood sample collection and biochemical analysis

Five milliliter (5 mL) of blood sample was collected from each study participant after overnight fasting with a serum separator tube. The sample was stored under an ice bag following blood drawing until transferred to National References Laboratory for Clinical Chemistry, EPHI for the analysis or stored at 2-8 °C until the analysis was done. The serum was obtained through centrifugation at 3000 rpm for 7 min using Rotanta 960 centrifuge in the thermo stable condition. The standard operational procedures in the pre-analytic, analytic and post-analytic steps of laboratory services were addressed to safeguard the overall quality of the laboratory analysis. Serum creatinine measurement was performed by an isotope dilution mass spectrometry (IDMS)-traceable enzymatic methods using Roche Modular Diagnostic, GmbH with intra- and inter-assay coefficients of variation of 0.9 and 2.9%, respectively. Then, eGFR was calculated using MDRD and CKD-EPI equations with an automatic calculator [11, 12]. The level of serum electrolytes were determined by the ion selective electrode principle of COBAS 6000 (c501) analyzer. Electrolyte measurements were done centrally. The level of circulating potassium was also determined from serum samples on Modular analyzer (Roche Diagnostics, Mannheim, Germany) with an inter-assay coefficient of variation of 1.4%.

The expected normal values of serum sodium, potassium and chloride were defined as; 135–145 mmol/L, 3.5–5.0 mmol/L and 98–107 mmol/L, respectively. Below this expected normal values were defined as hypo (hyponatremia; serum sodium level < 135 mmol/L, hypokalemia; serum potassium level < 3.5 mmol/L and hypochloremia; serum chloride level < 98 mmol/L), whereas above the expected values were defined as hyper (hypernatremia; serum sodium level > 145 mmol/L, hyperkalemia; serum potassium level > 5.0 mmol/L and hyperchloremia; serum chloride level > 107 mmol/L) [20]. Moreover, the expected normal values of serum calcium, phosphate and magnesium were defined as; 2.15–2.50 mmol/L [21], 0.18–1.45 mmol/L [22] and 0.66–1.07 mmol/L [21], respectively. Below their respective expected cut-off point of these electrolytes were defined as hypo (hypocalcemia; serum calcium level < 2.15 mmol/L, hypophosphatemia; serum phosphate level < 0.18 mmol/L and hypomagnesemia; serum magnesium level < 0.66 mmol/L) whereas above the expected normal values were defined as hyper (hypercalcemia; serum calcium level > 2.5 mmol/L, hyperphosphatemia; serum phosphate level > 1.45 mmol/L and hypermagnesemia; serum magnesium level > 1.07 mmol/L).

### Statistical analysis

Data from the biochemical analysis and questionnaires survey were recorded into Microsoft excel, then cleaned and transferred into a statistical package for the social science (SPSS) version 20 software for the statistical analysis. The descriptive data analysis were conducted and presented as frequency and percentage. We were also used a chi-square test to check the significance differences observed in eGFR category by the electrolyte level, and binary logistic regression analysis to determine the association of risk factors with reduced glomerular filtration rate. *P* value < 0.05 was used as a statistical significance.

### Data quality control

The quality of data was controlled at each phase of the study. A two-day training, which covers the entire activities of the study, was given for data collectors before running the data collection. To check the validity and reliability, the questionnaires and biochemical tests were pretested on 41 volunteer participants at St. Paul’s Hospital Millennium Medical College who had similar socio-demographic characteristics with the actual study participants. Thus, corrections and modifications were made according to the feedback we get from it. Data were also collected under a close supervision of the investigators. The biochemical test analysis was done by trained professional laboratory technologists, and the overall quality of the laboratory analysis was maintained through a strict follow-up of the manufacturer’s instruction and the standard operational procedure (SOP) in the pre-analytic, analytic and post analytic stages of the laboratory services. Besides, the biochemical test analysis was done at National References Laboratory for Clinical Chemistry, EPHI which is accredited and certified by Ethiopian National Accreditation Office (ENAO) with ISO 15189 requirements for medical laboratories. Moreover, hemolytic samples above the cut-off values were excluded from the study to control false values of the analytes. Data were double-checked for its completeness, consistency, accuracy and clarity on daily basis.

## Results

### Socio-demographic, behavioral and clinical characteristics of the study participants

Out of the total 412 study participants, the majority, 211/412 (51.2%) of them were males. Large proportion of the study participants, 169/412 (41.0%) were lied between the age of 18–32 years. About 18/412 (4.4%) were current smokers during the study period. The majority, 272/412 (66.0%) of the study participants were found to be alcohol consumers, while about 18/412 (4.4%) of the study participants were found to be current chat chewers. Only 6/412 (1.5%) of the participants had been used traditional healers for different health problems at least once in their lifetime before the study period (Table 1).

**Table 1:** Socio-demographic, behavioral and clinical characteristics of the study participants, Addis Ababa, Ethiopia, 2018 (*n* = 412)

Characteristics	Total (N)	(%)
Sex		
Male	211	51.2%
Female	201	48.8%
Age group		
18–32	169	41.0%
33–47	163	39.6%
≥ 48	80	19.4%
Marital status		
Never married	138	33.5%
Married	247	60.0%
separated, widowed, divorced	27	6.6%
Educational background		
no formal education	3	0.7%
primary education	75	18.2%
secondary education	63	15.3%
college & above	271	65.8%
Quartiles of income per month		
quartile1	104	25.2%
quartile2	102	24.8%
quartile3	103	25.0%
quartile4	103	25.0%
Smoking status		
Non smoker	372	90.3%
Former smoker	22	5.3%
Currently smoker	18	4.4%
Alcohol drinking status		
Yes	272	66.0%
No	140	34.0%
Chat chewing status		
Non chewer	347	84.2%
Former chewer	47	11.4%
Currently chewer	18	4.4%
Traditional medication		
Yes	6	1.5%
No	406	98.5%
History of hypertension		
Yes	74	18.0%
No	338	82.0%
History of diabetes		
Yes	14	3.4%
No	398	96.6%
History of CVD		
Yes	22	5.3%
No	390	94.7%
Previous kidney function test measurement		
Yes	157	38.1%
No	255	61.9%
Previous kidney function test abnormality		
Yes	48	30.6%
No	109	69.4%
History of kidney stone		
Yes	27	6.6%
No	385	93.4%
History of repeated UTI and/ or glomerulonephritis		
Yes	56	13.6%
No	356	86.4%
Family history of renal failure		
Yes	25	6.1%
No	387	93.9%

Quartile1 was less than 1500 birr, quartile2 = 1501–3173 birr, quartile3 = 3174–6676 birr, quartile4 was greater than 6676 birr. The former smoker and chewer were defined as those who smoke or chew before the time of data collection and stops during the study period. *UTI* Urinary tract infection, *CVD* Cardiovascular disease. Repeated UTI and/ or glomerulonephritis are defined as the history of more than three times per year exposure of UTI and/ or glomerulonephritis. Herbal medication was defined as participants who had ever taken any traditional healers throughout their life

Regarding BMI status of the participants, 132/412 (32.0%) and 35/412 (8.5%) of the participants were overweight and obese, respectively. About 68/412 (16.5%) and 87/412 (21.1%) by systolic and diastolic blood pressure were hypertensive during the study period, respectively. Concerning self-reported history of clinical illness, 74/412 (18%), 14/412 (3.4%) and 22/412 (5.3%) of the study participants had hypertension, diabetes and CVDs, respectively. Only 157/412 (38.1%) of the study participants had previous kidney function test checkup and aware of their kidney function status. About 27/412 (6.6%) and 25/412 (6.1%) of the study participants had self-reported history of kidney stone and family history of renal failure, respectively, whereas 56/412 (13.6%) had self-reported history of repeated urinary tract infection (UTI) and/or glomerulonephritis (Table 1).

### Prevalence of chronic kidney disease and staging

**Fig. 1:**
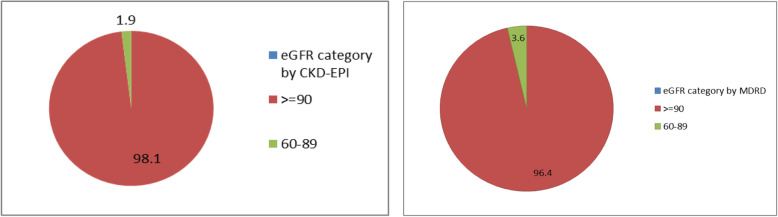
Pie chart to show the prevalence of CKD by MDRD and CKD-EPI equations of the study participants, Addis Ababa, Ethiopia, 2018 (*n* = 412)

Out of the total study participants, 3.6% (95% CI: 1.9–5.6%) and 1.9% (95% CI: 0.7–3.4%) were in stage II (eGFR between 60 and 89 ml/min/1.73m^2^) by MDRD and CKD-EPI equations, respectively. Besides, none of the study participants showed CKD (eGFR < 60 ml/min/1.73m^2^) regardless of the equations. The remaining participants which were 96.4% by MDRD and 98.1% by CKD-EPI equations had normal kidney function test or eGFR ≥90 ml/min/1.73m^2^ (Fig. 1).

### Distribution of eGFR category by electrolyte level

The majority of the study participants had normal electrolyte levels. Out of the total study participants, 2.4% hypernatremia, 0.5% hypokalemia, 9.5% hyperkalemia, 0.5% hypochloremia, 5.3% hyperchloremia, 8.5% hypocalcemia, 0.2% hypercalcemia, 0.7% hyperphosphatemia, 1.0% hypomagnesemia and 0.2% hypermagnesemia had shown an abnormalities in their serum electrolyte levels. Furthermore, none of the study participants showed hyponatremia and hypophosphatemia. Among hypocalcemia participants, 3/35 (8.6%) by MDRD and 2/35 (5.7%) by CKD-EPI equations were found to be a stage II kidney disease. About 1/3 (33.3%) by MDRD equation were in stage II among the hyperphosphatemia study participants (Table 2).

**Table 2:** Distribution of eGFR category using MDRD and CKD-EPI equations by electrolyte level, Addis Ababa, Ethiopia, 2018 (*n* = 412)

Serum electrolyte levels in mmol/L		Total participant N (%)	MDRD (GFR. ml/min/1.73 m^2^)			CKD-EPI (GFR. ml/min/1.73m^2^)		
			≥90 N (%)	60–89 N (%)	*P*-value	≥90 N (%)	60–90 N (%)	*P*-value
Sodium	Normal Hypernatremia	402 (97.6) 10 (2.4)	387 (96.3) 10 (100)	15 (3.7) 0 (0.0)	1.000	394 (98) 10 (100)	8 (2.0) 0 (0.0)	1.000
Potassium	Hypokalemia Normal Hyperkalemia	2 (0.5) 371 (90) 39 (9.5)	2 (100) 356 (96) 39 (100)	0 (0.0) 15 (4.0) 0 (0.0)	0.423	2 (100) 363 (97.8) 39 (100)	0 (0.0) 8 (2.2) 0 (0.0)	0.637
Chloride	Hypochloremia Normal Hyperchloremia	2 (0.5) 388 (94.2) 22 (5.3)	2 (100.0) 373(96.1) 22 (100)	0 (0.0) 15 (3.9) 0 (0.0)	0.618	2 (100) 380 (97.9) 22 (100)	0 (0.0) 8 (2.1) 0 (0.0)	0.777
Calcium	Hypocalcemia Normal Hypercalcemia	35 (8.5) 376 (91.3) 1 (0.2)	32 (91.4) 364 (96.8) 1 (100)	3 (8.6) 12 (3.2) 0 (0.0)	0.262	33 (94.3) 370 (98.4) 1 (100)	2 (5.7) 6 (1.6) 0 (0.0)	0.238
Phosphate	Normal Hyperphosphatemia	409 (99.3) 3 (0.7)	395(96.6) 2 (66.7)	14 (3.4) 1 (33.3)	0.106	401 (98.0) 3 (100)	8 (2.0) 0 (0.0)	1.000
Magnesium	Hypomagnesemia Normal Hypermagnesemia	4 (1.0) 407 (98.8) 1 (0.2)	4 (100) 392(96.3) 1 (100)	0 (0.0) 15 (3.7) 0 (0.0)	0.909	4 (100.0) 399 (98.0) 1 (100)	0 (0.0) 8 (2.0) 0 (0.0)	0.951

All serum electrolytes were measured through mmol/L. The normal value of each electrolyte level was as described on Cobase 6000 analyzer normal set point. *eGFR* Estimated glomerular filtration rate

### Associated factors of reduced GFR (< 90 ml/min/1.73m^2^)

We performed a multinomial logistic analysis for reduced GFR (< 90 ml/min/1.73m^2^) by MDRD equation using socio-demographic, behavioral and clinical characteristics of the study participants as covariates, and found that an older age (*P* = 0.006), high BMI (*P* = 0.045) and previous history of CVDs (*P* = 0.033) were statistically significant variables associated with reduced GFR (< 90 ml/min/1.73m^2^). However, a multinomial logistic analysis for reduced GFR by the CKD-EPI equation was not done as the number of study participants were very few with reduced GFR. Similarly, the association of an herbal medication use and repeated UTI/glomerulonephritis with stage II (G2) kidney disease was not analyzed since none of the study participants that used an herbal medication or diagnosed by repeated UTI/glomerulonephritis were under a stage II kidney disease (Table 3).

**Table 3:** Association of socio demographic, behavioral, and clinical characteristics with reduced GFR, Addis Ababa, Ethiopia, 2018 (*n* = 412)

Characteristics	MDRD		
	Distribution of reduced GFR (GFR < 90 ml/min/1.73m^2^)		
	N (%)	AOR (95% CI)	*P*-value
18–32	1 (0.6)	1	
33–47	5 (3.1)	3.435 (0.329–35.901)	0.303
≥48	9 (11.2)	32.325 (2.732–382.442)	0.006
Female	4 (2.0)	1	
Male	11 (5.2)	3.371 (0.656–17.325)	0.146
Single	3 (2.2)	1	
Married	11 (4.5)	0.838 (0.164–4.295)	0.832
Separated/Widowed	1 (3.7)	0.454 (.026–7.841)	0.587
Primary school &less	5 (6.7)	1	
Secondary	1 (1.6)	0.345(0.028–4.280)	0.408
>College &above	9 (3.3)	0.398 (0.061–2.599)	0.336
Quartile1	4 (3.8)	1	
Quartile2	1 (1.0)	0.100 (0.007–1.413)	0.088
Quartile3	5 (4.9)	0.660 (0.078–5.605)	0.703
Quartile4	5 (4.9)	0.613 (0.071–5.276)	0.656
Never smoked	12 (3.2)	1	
Smoker	3 (13.6)	0.744 (0.104–5.318)	0.768
Never drink alcohol	5 (3.6)	1	
Ever drink alcohol	10 (3.7)	0.593 (0.149–2.354)	0.458
Never chewed chat	11 (3.2)	1	
Chat chewer	4 (8.5)	1.374 (0.228–8.266)	0.728
BMI < 25 kg/m^2^	5 (2.0)	1	
≥ 25 kg/m^2^	10 (6.0)	4.088 (1.032–16.192)	0.045
Mean SBP < 140 mmHg	12 (3.4)	1	
≥140 mmHg	3 (4.8)	0.440 (0.063–3.055)	0.406
Mean DBP < 90 mmHg	13 (3.9)	1	
≥90 mmHg	2 (2.6)	0.387 (0.051–2.964)	0.361
History of HTN No	9 (2.7)	1	
Yes	6 (8.1)	2.243 (0.576–8.727)	0.244
History of diabetes No	14 (3.5)	1	
Yes	1 (7.1)	0.447 (0.036–5.555)	0.531
History of CVD No	13 (3.3)	1	
Yes	2 (9.1)	8.378 (1.194–58.795)	0.033
Kidney stone history No	13 (3.4)	1	
Yes	2 (7.4)	2.696 (0.425–17.112)	0.293
Family history of renal failure No	14 (3.6)	1	
Yes	1 (4.0)	1.788 (0.187–17.113)	0.614

*P*-value < 0.05 was used as statistically significant. *AOR* Adjusted odds ratio

## Discussion

The chronic kidney disease is one of the most common chronic NCDs that characterized by the persistent reduction of kidney functions with or without the structural abnormality of the kidney for more than 3 months [9, 10]. If CKD left untreated at early stage, it leads to complications like the electrolyte derangements, impairment of acid-base homeostasis, CVDs with its adverse effect, anemia, hyperuricaemia and CKD-MBD, which are even fetal [9, 23–25]. Globally, the incidence and prevalence increment of CKD is associated with different socio-demographic, behavioral and co-morbid conditions [5]. Out of them, hypertension and diabetes are the two risk independent and major causative factors of the kidney failure [26].

To the best of our knowledge, in Ethiopia, this is the first study on the prevalence of reduced GFR or CKD and its associated factors among the general population. Our target was to assess the association of risk factors with CKD (GFR < 60 ml/min/1.73m^2^), but none of the study participants was under CKD. Therefore, we assessed the association of the risk factors with stage II (G2) kidney disease (GFR < 90 ml/min/1.73m^2^). Among the total study participants, only 38.2% of them had been done kidney function tests in their life. This finding is in line with a study done in Kinshasa on high risk groups that reported, only 12% of the study participants were aware of their CKD before the study conducted, but the exact prevalence was found to be 36% during the study period [27]. The meta-analysis done in African countries also showed that very rare people were supposed to go for the screening of CKD; as a result, those who are aware of their kidney function status were too low [4]. Several meta-analysis reports also showed that the lack of awareness, attitude and practice towards a kidney disease, and its complications are the major obstacles to minimize the burden of the disease in developing countries [4, 6].

In this study, the prevalence of stage II kidney disease (eGFR between 60 and 89 ml/min/1.73m^2^) was 3.6% (95% CI: 1.9–5.6%) by MDRD and 1.9% (95% CI: 0.7–3.4%) by CKD-EPI equations. However, none of the study participants was found to be eGFR below 60 m/min/1.73m^2.^ This finding is far lower than a study done in India [28] that showed, the overall prevalence of CKD with 17.2 and 16.4% by MDRD and CKD-EPI equations, respectively. Conversely, stage II kidney disease (GFR < 90 ml/min/1.73m^2^) of the present finding is in line with the Indian study which reported 4.3 and 3.2% stage II kidney disease by MDRD and CKD-EPI equations, respectively. Our finding is also lower than another similar study done in Southern Iran [29] which showed that the overall prevalence of CKD (from stage II to V) was 11.6%, and the prevalence of stage I, II, III, IV and V kidney disease was 8.5, 66.1, 11.4, 0.1 and 0.1%, respectively by the MDRD equation but not reported by the CKD-EPI equation. The current finding is also far lower than another similar study done in Kinshasa (Democratic Republic of Congo) [27] by which the prevalence of CKD was found to be 36% in stage I, 6% in II, 18% in III, 2% in IV, and 6% in stage V by MDRD equation. The higher difference between these studies and the present study might be due to the genetic variation, variation of the validity of the estimators used and restriction of the study population. The main reason of getting lower prevalence of CKD in our study may be due to dietary factors (Ethiopians are mainly utilized traditional foods which may have a lower side effect for kidney damage) and institutional based study design. Moreover, meta-analysis results among Sub-Saharan Africa countries on CKD is also far higher than the present study [6]. However, the most difficult part to compare the finding of the present study with the meta-analysis done in Sub-Saharan Africa countries is an estimator variation since this meta-analysis was considered the C-G equation and proteinuria in addition to MDRD and CKD-EPI equations to determine the prevalence of CKD [6]. The finding of the current study is also far lower than a study done in Southern Ethiopia on selected diabetic patients [30]. Even though this study was done in the same country, having the same cultural background, the major result difference with the current study might be due to the restriction of the study population and difference of the estimators used. The study done in Southern Ethiopia focused on selected diabetic patients.

On the contrary, our finding is similar to a study done in Chennai India for the screening of CKD in the general population which reported a decreased prevalence of kidney function test (eGFR < 80 ml/min.m^2^) between 0.86 to 1.39% by the C-G equation [31]. The present study did not consider the variation of the cut-off points used for reduced filtration rate and the variation of estimators. However, the prevalence of CKD and its staging can be affected even within a homogenous population depending on the estimators used [32]. Several reports have revealed that the CKD-EPI equation is the most recent and lowest limitations compared to the C-G and MDRD equations [9]. Although CKD was not found in the present study, study participants with a stage II will have high risk of getting renal failure over time. In support of this, a perspective follow-up study done on the kidney disease [33] showed that 1.1% of a stage II participants developed an end stage renal failure over 5 years and required dialysis or kidney transplantation.

Most studies done to screen for electrolyte derangements have been focused among hospitalized patients and rarely done in the general populations. This makes difficult to compare and contrast electrolyte disorder in the community. In our study, the most common electrolyte derangement encountered were hyperkalemia (9.5%) and hypocalcemia (8.5%). This is in contrast to a study done in Netherland [34] which revealed that hyponatremia (7.7%) and hypernatremia (3.4%) were the most common electrolyte derangements. This may be due to the restriction of age in the study participants since the Netherlands researcher was focused on the older age groups above 55 years. Besides, no one was under hyponatremia in this study unlike that of the Netherland’s study. This may be due to a high amount of sodium intake as salt in our study participants. The Ethiopian national survey on a salt intake also showed that the mean dietary salt intake of the Ethiopian population (8.3 g/day) [35] is far higher than the World Health Organization (WHO) recommended daily salt intake (should be less than 5 g/day) [36]. The prevalence of hyperkalemia (9.5%) in this study is also much higher than another study in subjects from the general population (age 28–75) which reports 3.8% of hyperkalemia prevalence [37]. This variation may be due to the ethnic background difference. In this study, hypercalcaemia (0.2%) was far lower than hypocalcemia (8.5%). In line with this, the incidence of hypercalcaemia (4.7%) was lower than hypocalcemia (27.7%) in hospitalized patients [38]. Our finding is also supported by another review [39] that showed hypercalcemia is less common than hypocalcemia in the general population. Another study also showed that hypercalcemia can be manifested up to 1% in the general population [40] which is comparable to the present study.

Most of the study participants with electrolyte derangements except hyperphosphatemia and hypocalcemia had normal GFR (GFR ≥ 90 ml/min/1.73m^2^) by both estimators. About 8.6 and 5.7% of hypocalcemia participants were stage II kidney diseases by MDRD and CKD-EPI equations, respectively. One of the study participants from the hyperphosphatemia was stage II kidney disease (GFR < 90 ml/min/1.73m^2^). Even though there are limited number of studies on the relationship between the electrolyte derangement and kidney function test in the general population, CKD development is one of the major cause of the electrolyte derangements [17]. Electrolyte disorders in CKD is one of the most common indication of kidney tubular damage and become common when CKD transforms into ESRF [9]. In our study, none of the study participants was under serious kidney disease and the result showed that the electrolyte derangements were less likely to be associated with reduced glomerular filtration rate.

In this study, an older age, high BMI and previous history of CVDs were the factors found to be significantly associated with stage II kidney disease (*P* = 0.006), (*P* = 0.045) and (*P* = 0.033), respectively. The association of an older age with reduced glomerular filtration rate is supported by other studies done in South West Nigeria [41], India [28] and China [42]. On the other hand, this association is on the contrary to the study done in South Africa [43] which revealed that high prevalence of CKD was found in the younger age group between 20 and 50 years than the older age groups. The meta-analysis on Sub-Saharan countries population on the prevalence and associated factors of CKD also showed that the younger age group was the most affected groups with CKD which makes it more serious in this area [6]. This variation might be due to the increment of other risk factors like hypertension, diabetes mellitus and retroviral infection during young age.

In the current finding, a high level of BMI which indicates the obesity status of the population was found to be one of the contributing factors of reduced glomerular filtration rate. This is supported by similar studies done in Saudi [44], Southern Iran [29] and China [42]. One systematic review also reported that a strong positive association between weight loss and improvement of kidney function tests is commonly encountered [45]. The exact molecular mechanism of obesity related renal injury or dysfunction is not well understood yet, but supposed to be due to a change of hemodynamic stability, metabolic disorder, activation of inflammatory process and glomerular hyper filtration [46–48]. Even though obesity can be contributed for the development of CKD through the progression of hypertension and diabetes, the two major causative factors of CKD, there are also evidences that confirmed hypertension or diabetes independent association of obesity with a renal dysfunction/CKD [49]. A renal dysfunction can be directly caused due to the accumulation of ectopic fats in and around the kidney [48]. During obesity, ectopic lipid accumulates in non-adipose tissues, including kidney cells [49]. This leads to an excessive production of toxic lipid metabolites such as ceramides and diacylglycerol in non-adipose tissue cells. These toxic metabolites further lead to the renal dysfunction or injury through the mitochondrial dysfunction and endoplasmic reticulum stress pathway [50].

Another associated factor with reduced glomerular filtration rate in our study was previous history of CVDs. This is in line with a systematic review done by Kazory and Ross, Gajjala et al, Schefold et al, and Elhafeez et al [4, 51, 52] which showed that CKD and CVD are interdependent to each other (CKD is risk factor for CVD and the reverse is also true). The CKD increases the risk of getting CVD by 2–4 times in the general population [53]. While the exact molecular mechanism is not well understood yet, some of the metabolic pathways that interrelate CVD and CKD are supposed to be key factor for these interrelationship activities. Some of these pathways are hyperactivity of RAAS, inflammation, osmotic sodium retention, endothelial dysfunction, dyslipidemia, and phosphatidylinositol 3-kinase (PI 3-kinase)-dependent signaling pathways [51]. In addition, CKD and CVD share common risk factors such as smoking, obesity, hypertension, diabetes mellitus and dyslipidemia which may be contributed for the occurrence of CVD along with CKD.

### Strength and limitation of the study

The study was focused on the screening of the participants for CKD and electrolytes, which is not commonly practiced in our country, was the main strength of the study, whereas an institutional based study which did not show the exact prevalence and burden of CKD in the general population of Ethiopia, was the main limitation of the study. A cross-sectional study design, which did not show the exact cause and effect relationship of the disease and factors, was also another limitation of the study. We were also unable to classify stage I CKD because we did not measure proteinuria due to reagent unavailability during the study period.

## Conclusions

In this cross-sectional study of screening for the early detection and identification of CKD, we found that 3.6 and 1.9% stage II kidney disease by MDRD and CKD-EPI equations, respectively. None of the study participants was under critical kidney disease or GFR < 60 mmol/min/1.73m^2^. An older age, high BMI and previous history of CVDs were significantly associated with reduced glomerular filtration rate. Even though the prevalence of the kidney disease was relatively low, an early detection and screening of CKD should be practiced at the primary health care level in order to prevent and minimize an end stage renal failure.

## Data Availability

The datasets used and/or analysed during the current study are available from the corresponding author on reasonable request.

## Abbreviations

CKD: Chronic kidney diseases
GFR: Glomerular filtration rate
eGFR: Estimated glomerular filtration rate
ESRD: End-stage renal disease
C-G: Cock croft – gault equation
MDRD: Modification of diet in renal disease
CKD-EPI: Chronic kidney disease epidemiology collaboration
KDIGO: Kidney disease improving global outcome
CVD: Cardiovascular disease

## References

[1] Lozano R, Naghavi M, Foreman K, Lim S, Shibuya K, Aboyans V (2012). Global and regional mortality from 235 causes of death for 20 age groups in 1990 and 2010: a systematic analysis for the global burden of disease study 2010. Lancet.

[2] Naghavi M, Abajobir AA, Abbafati C, Abbas KM, Abd-Allah F, Abera SF (2017). Global, regional, and national age-sex specific mortality for 264 causes of death, 1980–2016: a systematic analysis for the global burden of disease study 2016. Lancet.

[3] Topf JM, Inker LA. MEASUREMENT OF GLOMERULAR FILTRATION RATE. Nephrology Secrets: First South Asia Edition-E-Book 2018:22.

[4] ElHafeez SA, Bolignano D, D’Arrigo G, Dounousi E, Tripepi G, Zoccali C (2018). Prevalence and burden of chronic kidney disease among the general population and high-risk groups in Africa: a systematic review. BMJ Open.

[5] Hill NR, Fatoba ST, Oke JL, Hirst JA, O’Callaghan CA, Lasserson DS (2016). Global prevalence of chronic kidney disease–a systematic review and meta-analysis. PLoS One.

[6] Stanifer JW, Jing B, Tolan S, Helmke N, Mukerjee R, Naicker S (2014). The epidemiology of chronic kidney disease in sub-Saharan Africa: a systematic review and meta-analysis. Lancet Glob Health.

[7] Arogundade FA, Barsoum RS (2008). CKD prevention in sub-Saharan Africa: a call for governmental, nongovernmental, and community support. Am J Kidney Dis.

[8] Baumgarten M, Gehr T (2011). Chronic kidney disease: detection and evaluation. Am Fam Physician.

[9] Levin A, Stevens PE, Bilous RW, Coresh J, De Francisco AL, De Jong PE (2013). Kidney disease: improving global outcomes (KDIGO) CKD work group. KDIGO 2012 clinical practice guideline for the evaluation and management of chronic kidney disease. Kidney Int Suppl.

[10] Webster AC, Nagler EV, Morton RL, Masson P (2017). Chronic kidney disease. Lancet.

[11] Levey AS, Bosch JP, Lewis JB, Greene T, Rogers N, Roth D (1999). A more accurate method to estimate glomerular filtration rate from serum creatinine: a new prediction equation. Ann Intern Med.

[12] Levey AS, Stevens LA, Schmid CH, Zhang YL, Castro AF, Feldman HI (2009). A new equation to estimate glomerular filtration rate. Ann Intern Med.

[13] Alcázar RA (2008). Electrolyte and acid-base balance disorders in advanced chronic kidney disease. Nefrologia.

[14] Schrier RW. Renal and electrolyte disorders: Lippincott Williams & Wilkins; 2010..

[15] Luo J, Brunelli SM, Jensen DE, Yang A (2016). Association between serum potassium and outcomes in patients with reduced kidney function. Clin J Am Soc Nephrol.

[16] Levin A, Bakris G, Molitch M, Smulders M, Tian J, Williams L (2007). Prevalence of abnormal serum vitamin D, PTH, calcium, and phosphorus in patients with chronic kidney disease: results of the study to evaluate early kidney disease. Kidney Int.

[17] Dhondup T, Qian Q (2017). Electrolyte and acid-base disorders in chronic kidney disease and end-stage kidney failure. Blood Purif.

[18] Organization WH, Organization WH, Organization WH, Organization WH. Defining the problem of overweight and obesity. World Health Organization Obesity: preventing and managing the global epidemic: report of a Who Consultation Geneva 2000:241–243. 11234459

[19] Guideline S (1999). 1999 World Health Organization-International Society of Hypertension guidelines for the management of hypertension. J Hypertens.

[20] Burtis CA, Bruns DE. Tietz fundamentals of clinical chemistry and molecular diagnostics-E-book: Elsevier health sciences; 2014.

[21] Wu AH. Tietz clinical guide to laboratory tests-E-book: Elsevier health sciences; 2006.

[22] Burtis CA, Ashwood ER, Bruns DE. Tietz textbook of clinical chemistry and molecular diagnostics-e-bookes: Elsevier health sciences; 2012.

[23] Cirillo M, Laurenzi M, Mancini M, Zanchetti A, Lombardi C, De Santo N (2006). Low glomerular filtration in the population: prevalence, associated disorders, and awareness. Kidney Int.

[24] Romagnani P, Remuzzi G, Glassock R, Levin A, Jager KJ, Tonelli M (2017). Chronic kidney disease. Nat Rev Dis Primers.

[25] Thomas B, Matsushita K, Abate KH, Al-Aly Z, Ärnlöv J, Asayama K (2017). Global cardiovascular and renal outcomes of reduced GFR. J Am Soc Nephrol.

[26] Chen RA, Scott S, Mattern WD, Mohini R, Nissenson AR (2006). The case for disease management in chronic kidney disease. Dis Manag.

[27] Sumaili EK, Cohen EP, Zinga CV, Krzesinski J-M, Pakasa NM, Nseka NM (2009). High prevalence of undiagnosed chronic kidney disease among at-risk population in Kinshasa, the Democratic Republic of Congo. BMC Nephrol.

[28] Singh AK, Farag YM, Mittal BV, Subramanian KK, Reddy SRK, Acharya VN (2013). Epidemiology and risk factors of chronic kidney disease in India–results from the SEEK (screening and early evaluation of kidney disease) study. BMC Nephrol.

[29] Khajehdehi P, Malekmakan L, Pakfetrat M, Roozbeh J, Sayadi M (2014). Prevalence of chronic kidney disease and its contributing risk factors in southern Iran: a cross-sectional adult population-based study. Iran J Kidney Dis.

[30] Fiseha T, Kassim M, Yemane T (2014). Prevalence of chronic kidney disease and associated risk factors among diabetic patients in southern Ethiopia. Am J Health Res.

[31] Mani MK (2005). Experience with a program for prevention of chronic renal failure in India. Kidney Int.

[32] Earley A, Miskulin D, Lamb EJ, Levey AS, Uhlig K (2012). Estimating equations for glomerular filtration rate in the era of creatinine standardization: a systematic review. Ann Intern Med.

[33] Keith DS, Nichols GA, Gullion CM, Brown JB, Smith DH (2004). Longitudinal follow-up and outcomes among a population with chronic kidney disease in a large managed care organization. Arch Intern Med.

[34] Liamis G, Rodenburg EM, Hofman A, Zietse R, Stricker BH, Hoorn EJ (2013). Electrolyte disorders in community subjects: prevalence and risk factors. Am J Med.

[35] Challa F, Tadesse Y, Mudie K, Taye G, Gelibo T, Bekele A (2017). Urinary sodium excretion and determinates among adults in Ethiopia: findings from national STEPS survey. Ethiop J Health Dev.

[36] WHO. Guideline: Sodium intake for adults and children: World Health Organization; 2012. 23658998

[37] Kieneker LM, Eisenga MF, Joosten MM, de Boer RA, Gansevoort RT, Kootstra-Ros JE (2017). Plasma potassium, diuretic use and risk of developing chronic kidney disease in a predominantly white population. PLoS One.

[38] Catalano A, Chilà D, Bellone F, Nicocia G, Martino G, Loddo I, Morabito N, Benvenga S, Loddo S (2018). Incidence of hypocalcemia and hypercalcemia in hospitalized patients: Is it changing?. J Clin Transl Endocrinol..

[39] Wang TJ, Zhang F, Richards JB, Kestenbaum B, Van Meurs JB, Berry D (2010). Common genetic determinants of vitamin D insufficiency: a genome-wide association study. Lancet.

[40] Frølich A (1998). Prevalence of hypercalcaemia in normal and in hospital populations. Dan Med Bull.

[41] Oluyombo R, Ayodele O, Akinwusi P, Okunola O, Akinsola A, Arogundade F (2013). A community study of the prevalence, risk factors and pattern of chronic kidney disease in Osun state, south West Nigeria. West Afr J Med.

[42] Zhang L, Wang Z, Chen Y, Wang X, Chen Z, Feng B (2018). Prevalence and risk factors associated with chronic kidney disease in adults living in 3 different altitude regions in the Tibetan plateau. Clin Chim Acta.

[43] Matsha TE, Yako YY, Rensburg MA, Hassan MS, Kengne AP, Erasmus RT (2013). Chronic kidney diseases in mixed ancestry south African populations: prevalence, determinants and concordance between kidney function estimators. BMC Nephrol.

[44] Emem-Chioma P, Siminialayi I, Wokoma F (2011). Prevalence of chronic kidney disease in adults with metabolic syndrome. Saudi J Kidney Dis Transplant.

[45] Afshinnia F, Wilt TJ, Duval S, Esmaeili A, Ibrahim HN (2009). Weight loss and proteinuria: systematic review of clinical trials and comparative cohorts. Nephrol Dial Transplant.

[46] Hall JE, Crook ED, Jones DW, Wofford MR, Dubbert PM (2002). Mechanisms of obesity-associated cardiovascular and renal disease. Am J Med Sci.

[47] Slee AD (2012). Exploring metabolic dysfunction in chronic kidney disease. Nutr Metab.

[48] Hall ME, do Carmo JM, da Silva AA, Juncos LA, Wang Z, Hall JE (2014). Obesity, hypertension, and chronic kidney disease. Int J Nephrol Renovasc Dis.

[49] Ejerblad E, Fored CM, Lindblad P, Fryzek J, McLaughlin JK, Nyrén O (2006). Obesity and risk for chronic renal failure. J Am Soc Nephrol.

[50] Unger RH, Scherer PE (2010). Gluttony, sloth and the metabolic syndrome: a roadmap to lipotoxicity. Trends Endocrinol Metab.

[51] Gajjala PR, Sanati M, Jankowski J (2015). Cellular and molecular mechanisms of chronic kidney disease with diabetes mellitus and cardiovascular diseases as its comorbidities. Front Immunol.

[52] Schefold JC, Filippatos G, Hasenfuss G, Anker SD, Von Haehling S (2016). Heart failure and kidney dysfunction: epidemiology, mechanisms and management. Nat Rev Nephrol.

[53] Gansevoort RT, Correa-Rotter R, Hemmelgarn BR, Jafar TH, Heerspink HJL, Mann JF (2013). Chronic kidney disease and cardiovascular risk: epidemiology, mechanisms, and prevention. Lancet.

